# Design and Development of a Compact, Portable Nondispersive Infrared (NDIR)-Based Capnography Device for Real-Time End-Tidal Carbon Dioxide (CO₂) Monitoring

**DOI:** 10.7759/cureus.97324

**Published:** 2025-11-20

**Authors:** Drin Rrmoku

**Affiliations:** 1 Electrical Engineering, Independent Practice, Prishtina, ALB

**Keywords:** acute hypoxemic respiratory failure, capnography, carbon dioxide measurement, end-tidal carbon dioxide, healthcare diagnostics technology, portable medical device, research in emergency medicine, respiratory monitoring

## Abstract

Measuring carbon dioxide (CO₂) in exhaled breath provides critical insight into both ventilation and metabolism. Changes in CO₂ levels can indicate hypoventilation, airway obstruction, circulatory compromise, or metabolic imbalance, allowing the early detection of respiratory distress and other life-threatening conditions. Capnography provides continuous, non-invasive monitoring of respiratory status and metabolic function through the measurement of end-tidal CO₂ (ETCO₂). This work focuses on developing a low-cost, portable, battery-powered capnograph that enables accurate, continuous CO₂ monitoring with minimal discomfort during daily activities.

This study presents the design of a portable, battery-powered capnograph for non-invasive monitoring, featuring high measurement precision and a lightweight form factor. The device integrates a rechargeable power system and a Bluetooth wireless interface to enable seamless data transmission to healthcare providers. Signal acquisition and CO₂ concentration estimation are processed in real time using an embedded microcontroller.

The prototype successfully performs real-time CO₂ monitoring and wireless data transmission. Based on projected power consumption, the system enables a continuous runtime of up to approximately 22 hours on a 2000 mAh lithium battery, depending on usage conditions. Expected sensing performance follows the SprintIR-6S specifications, supporting measurements up to 20% CO₂ concentration within specified accuracy limits.

The compact, portable design supports reliable continuous monitoring and may enhance the early detection of respiratory deterioration in both clinical and emergency settings. By enabling the rapid recognition of changes in ventilatory status, such a system has the potential to improve patient outcomes and expand access to timely respiratory care.

## Introduction

Respiratory diseases represent a growing global health challenge. In 2019, chronic respiratory diseases (CRDs) affected approximately 454.6 million people worldwide and caused about four million deaths, with absolute case numbers rising significantly since 1990 [1,2]. Among these, conditions such as asthma and chronic obstructive pulmonary disease (COPD) constitute large proportions of the disease burden [3]. The economic toll is also high; COPD alone is projected to cost the world economy INT$4.326 trillion between 2020 and 2050 [4], and US COPD-related medical costs reached approximately US$31.3 billion in 2019, with growth toward US$60.5 billion by 2029 [5]. Early detection of respiratory deterioration is essential for preventing adverse outcomes, particularly in pre-hospital, emergency medicine, and home-care settings where access to advanced monitoring equipment is limited. Measurement of carbon dioxide (CO₂) in exhaled breath supports the continuous assessment of respiratory function, providing insight into ventilation effectiveness, airway patency, and circulatory status [6]. End-tidal CO₂ (ETCO₂) monitoring can indicate hypoventilation, airway obstruction, ventilation-perfusion mismatch, or circulatory compromise, enabling the timely recognition of life-threatening events [7]. To meet the increasing need for accessible respiratory monitoring, capnography offers a non-invasive method to continuously track ETCO₂ and display the waveform of exhaled gas, making it a valuable diagnostic and safety tool in both clinical and field environments [8,9].

Currently, there are two types of capnographs: the "sidestream" and the "mainstream" [10]. In mainstream systems, the CO₂ sensor is placed directly in the airway so that exhaled gas is measured immediately as the patient breathes out. In sidestream systems, a nasal cannula or small sampling tube continuously draws a tiny flow of exhaled air from the patient to a sensor located inside the monitoring device, allowing comfortable, non-invasive measurement without attaching hardware to the airway. Most capnographs use nondispersive infrared (NDIR) sensing to detect CO₂ levels, while advanced systems may use techniques such as Raman or photoacoustic spectroscopy [10].

The capnograph has a square-wave pattern [11]. In Phase I (baseline), at the start of exhalation, air from the upper airway is released, containing almost no CO₂, so the waveform remains at baseline. In Phase II (upstroke), as exhalation continues, CO₂-rich air from the lungs begins to mix in, causing a rapid rise in the waveform. In Phase III (alveolar plateau), mostly alveolar gas is exhaled, CO₂ levels stabilize, and this flat region represents the patient's true ETCO₂. In Phase IV (inspiration), when inhalation begins, fresh air enters the lungs and the waveform quickly drops back to zero CO₂.

To address the limitations of conventional capnography systems, which are often expensive, bulky, and primarily restricted to hospital environments, this work presents the design of a compact, low-cost portable capnograph intended for continuous use in real-world conditions. The system integrates a lightweight enclosure, rechargeable battery power, wireless data transmission, and a high-performance NDIR CO₂ sensor to support accurate ETCO₂ monitoring during daily activities, patient transport, and pre-hospital care. By minimizing size and power consumption while maintaining measurement precision, this device aims to expand access to respiratory monitoring for patients at risk of deterioration in both emergency and home-care settings.

## Technical report

Design approach

**Figure 1:**
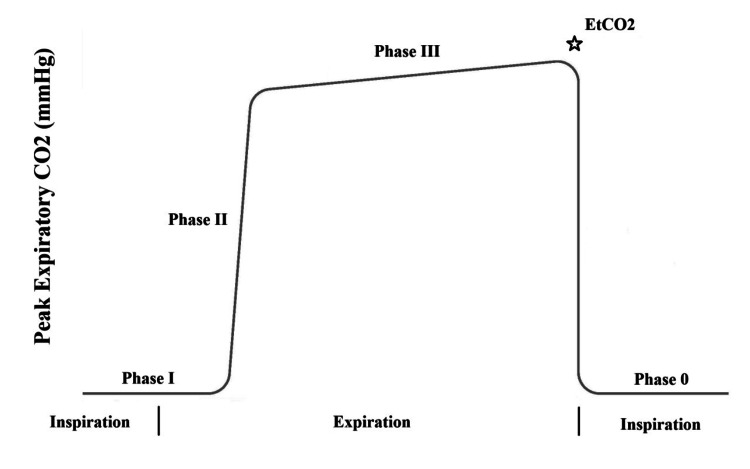
Normal capnography waveform showing the four respiratory phases This waveform illustrates the typical square-shaped capnography pattern during a single respiratory cycle. CO₂: carbon dioxide; ETCO₂: end-tidal carbon dioxide Image Credit: Author's original illustration

The proposed portable capnograph was developed with a focus on low cost, compact size, and reliable respiratory monitoring during daily activities and pre-hospital response. The system architecture includes an NDIR CO₂ sensor for gas measurement, an ESP32 microcontroller for signal processing and wireless communication, and a rechargeable lithium battery powering all subsystems. The overall design emphasizes low power consumption while maintaining accurate ETCO₂ estimation (Figure 1).

A SprintIR-6S NDIR (Gas Sensing Solutions, UK) sensor was selected due to its fast response time and capability to measure CO₂ concentrations up to 20%, supporting real-time waveform acquisition during every breath. Exhaled gas is delivered to the sensor chamber through a short nasal cannula line, minimizing dead-space effects. A small DC motor pump is activated only briefly before measurement cycles to clear residual gas and refresh the sensing region, improving reading stability.

Signal acquisition and data processing are handled by the ESP32, which performs real-time waveform analysis and transmits ETCO₂ values via Bluetooth for wireless monitoring. The power system is regulated by TPS63070 buck-boost converters to ensure stable operation over the full battery discharge range. Mechanical design considerations included device miniaturization, ease of patient use, and unobtrusive integration for both emergency personnel and home-care environments.

**Figure 2:**
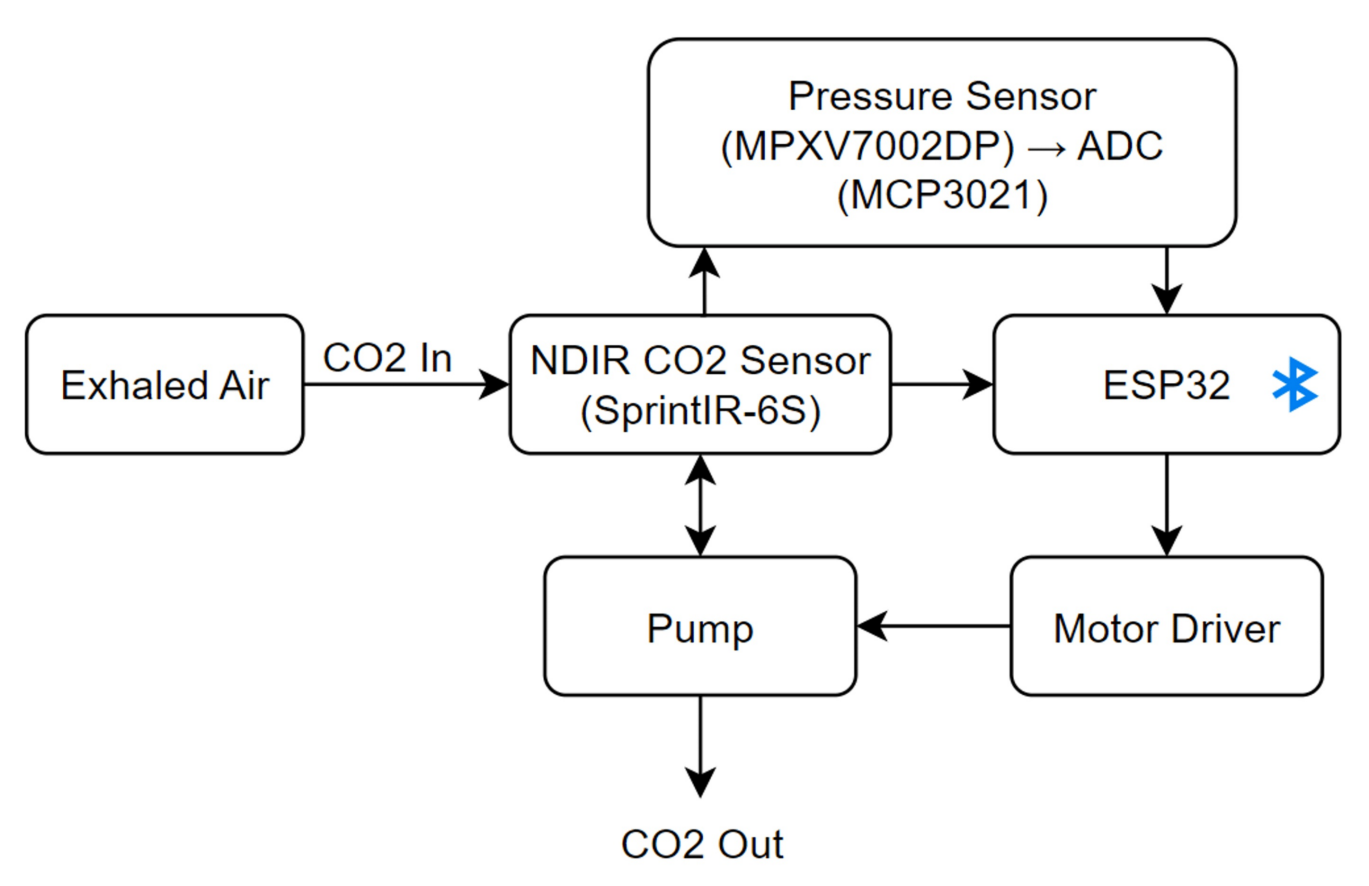
System block diagram of the portable capnograph device The ESP32 microcontroller processes CO₂ and pressure sensor signals while controlling the miniature pump to clear residual CO₂ between breaths. Wireless connectivity enables real-time respiration monitoring. CO₂: carbon dioxide Image Credit: Author's original illustration

Figure 2 shows the system architecture of the developed capnograph. Exhaled air is directed through a sensing module that measures CO₂ concentration and airflow pressure. The collected data are processed by a microcontroller, which also controls the pump and transmits the results wirelessly.

Hardware components

The device consists of five primary hardware modules: an NDIR CO₂ sensing unit, a microcontroller with integrated wireless capability, a digital acquisition interface, power management circuitry, and a compact enclosure designed for wearable or handheld use.

CO₂ Sensing Unit

A SprintIR-6S NDIR sensor was selected due to its rapid response time, high sensitivity, and capability of measuring CO₂ concentrations of up to 20%. The sensor operates by measuring infrared light absorption at specific wavelengths corresponding to CO₂, enabling the continuous acquisition of the capnographic waveform.

Microcontroller and Wireless Communication

An ESP32-C3 module (Espressif Systems, China) manages device control, serial communication with the CO₂ sensor, and wireless Bluetooth data transmission. Its low-power operation and integrated Wi-Fi/BLE support facilitate real-time remote monitoring.

Signal Conditioning and Data Conversion

An MCP3021 10-bit ADC (Microchip Technology) is used to digitize sensor output signals when analog interfacing is required. The interface ensures reliable signal integrity for CO₂ concentration reconstruction and waveform visualization.

Power Management Circuitry

Two TPS63070 buck-boost regulators (Texas Instruments, USA) generate isolated 3.3 V and 4.5 V rails from a single lithium battery input. Their high-efficiency conversion maintains stable performance across the full battery discharge range. A rechargeable lithium cell powers the system, providing an estimated 22 hours of operation depending on sampling frequency and pump activation cycles.

Airflow Control Mechanism

A miniature DC motor-driven pump is activated briefly prior to measurement cycles to replenish the sensing chamber with fresh exhaled gas, improving repeatability and measurement accuracy.

**Figure 3:**
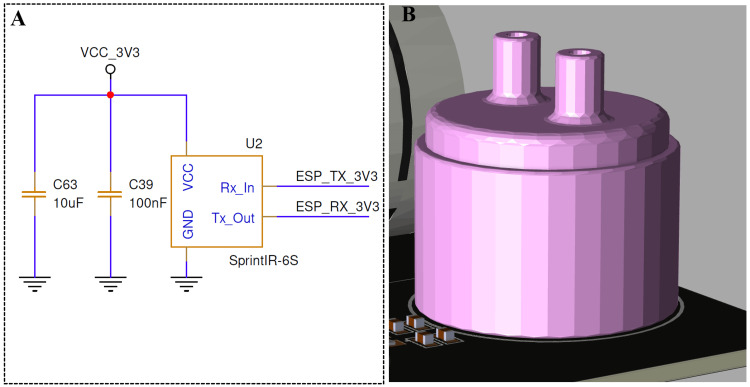
Electrical interface and mechanical integration of the NDIR CO₂ sensor (A) The UART interface circuitry for the SprintIR-6S NDIR CO₂ sensor, including 10 μF and 100 nF decoupling capacitors and serial communication links to the ESP32 microcontroller. (B) The 3D mechanical model of the sensor mounted on the PCB, highlighting the dual airway ports for gas sampling. CO₂: carbon dioxide; NDIR: nondispersive infrared; PCB: printed circuit board; UART: universal asynchronous receiver/transmitter Image Credit: Author's original illustration

To integrate real-time CO₂ measurement into the system, the SprintIR-6S NDIR sensor was integrated as shown in Figure 3. The sensor is powered from a regulated 3.3 V supply with local decoupling using 10 μF and 100 nF capacitors to ensure supply stability and noise reduction during high-speed sampling. The digital TX/RX lines provide bidirectional communication for transmitting measured CO₂ concentration values and receiving configuration commands.

**Figure 4:**
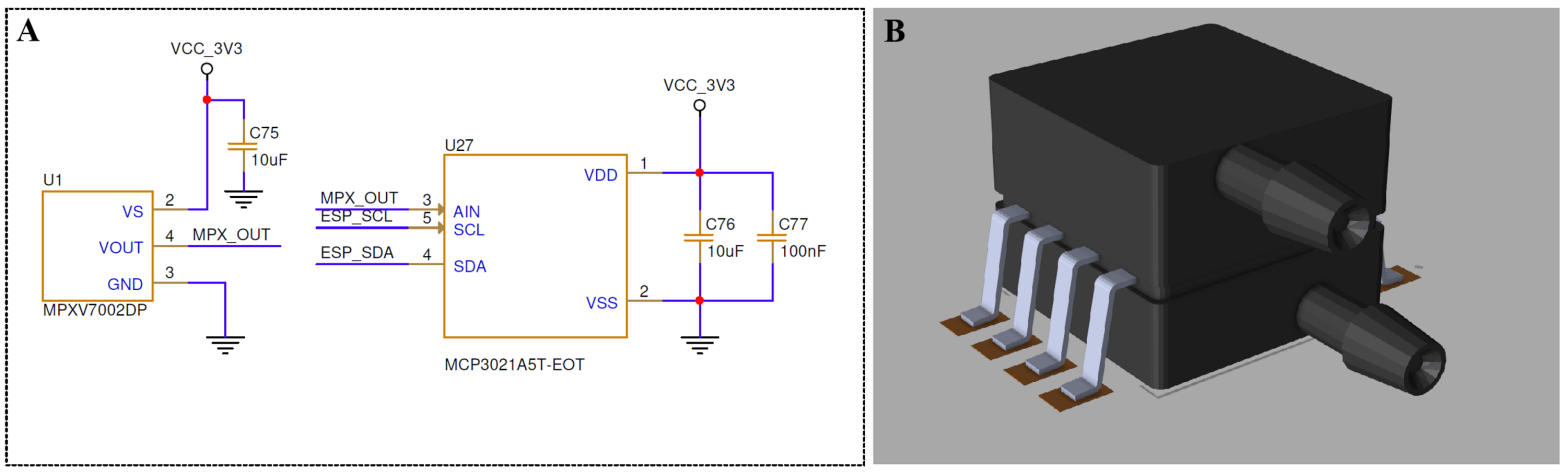
Pressure sensing and analog front-end for respiration phase detection (A) The signal conditioning circuitry for the MPXV7002DP differential pressure sensor used to detect airflow during both inspiration and expiration. The analog voltage output (MPX_OUT) is routed to an MCP3021 ADC for digitization over the I²C interface with the ESP32 microcontroller. (B) The 3D mechanical model of the differential sensor mounted to the PCB. PCB: printed circuit board Image Credit: Author's original illustration

To determine the respiratory phase and ensure accurate ETCO₂ waveform alignment, airflow pressure was measured using an MPXV7002DP differential pressure sensor placed in line with the sampling tubing. The sensor produces an analog voltage proportional to the pressure differential between the inhalation and exhalation ports, enabling the detection of breath initiation and end-tidal transitions. As shown in Figure 4, the sensor output is digitized using an MCP3021 10-bit ADC communicating with the ESP32 microcontroller over inter-integrated circuit (I²C), allowing synchronized sampling with the CO₂ sensor.

**Figure 5:**
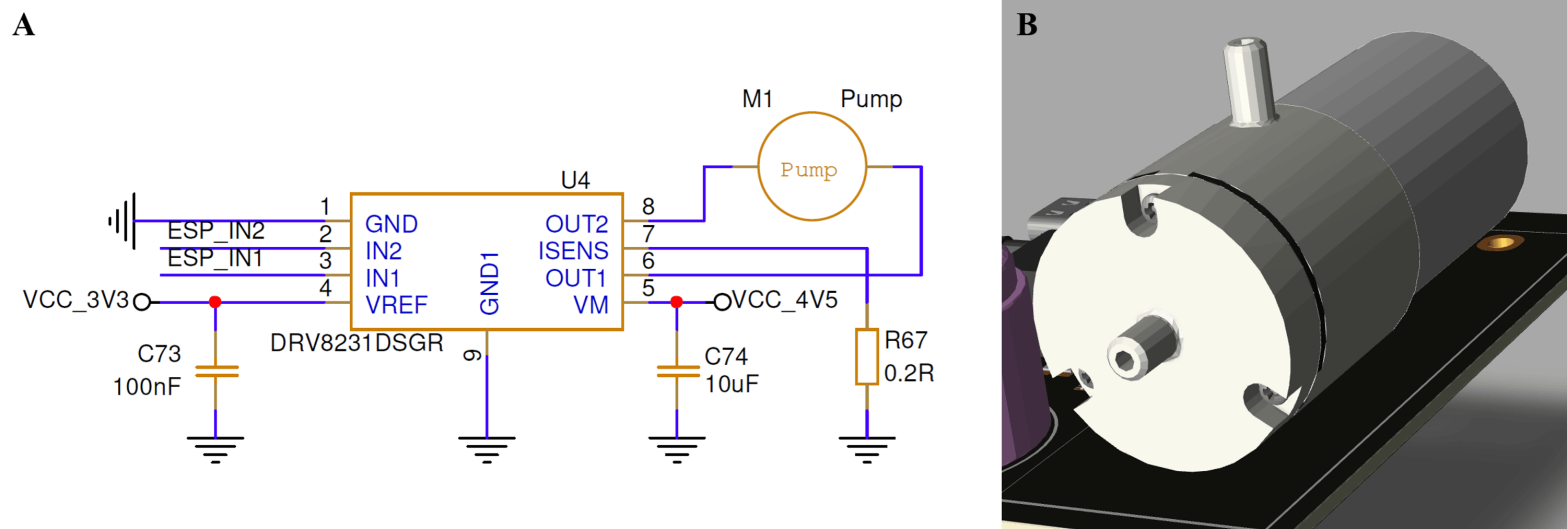
Miniature pump drive circuit and mechanical integration for CO₂ chamber purging (A) The DRV8231 motor driver circuitry used to control the pump from the ESP32 microcontroller. The motor driver receives logic-level signals (ESP_IN1/IN2) and provides current-controlled drive to the pump motor at a regulated 4.5 V supply. A 0.2 Ω sense resistor monitors motor current, while local decoupling capacitors suppress switching noise and ensure stable operation during rapid activation cycles. (B) The 3D mechanical model of the miniature diaphragm pump mounted to the PCB, responsible for evacuating residual CO₂ from the sampling chamber between breaths. CO₂: carbon dioxide; PCB: printed circuit board Image Credit: Author's original illustration

The diaphragm pump control stage was designed to ensure the rapid evacuation of residual CO₂ from the sampling chamber after each breath cycle. This prevents gas buildup and ensures the NDIR sensor baseline returns to zero before the next measurement, maintaining signal accuracy. The pump is driven by a DRV8231 motor driver, which allows full H-bridge control from two GPIO pins of the ESP32 and provides current feedback for fault detection. The design minimizes switching noise through local decoupling and enables precise timing control through the firmware. Figure 5 illustrates both the motor driver circuit and the mechanical layout of the miniature diaphragm pump used in the prototype.

**Figure 6:**
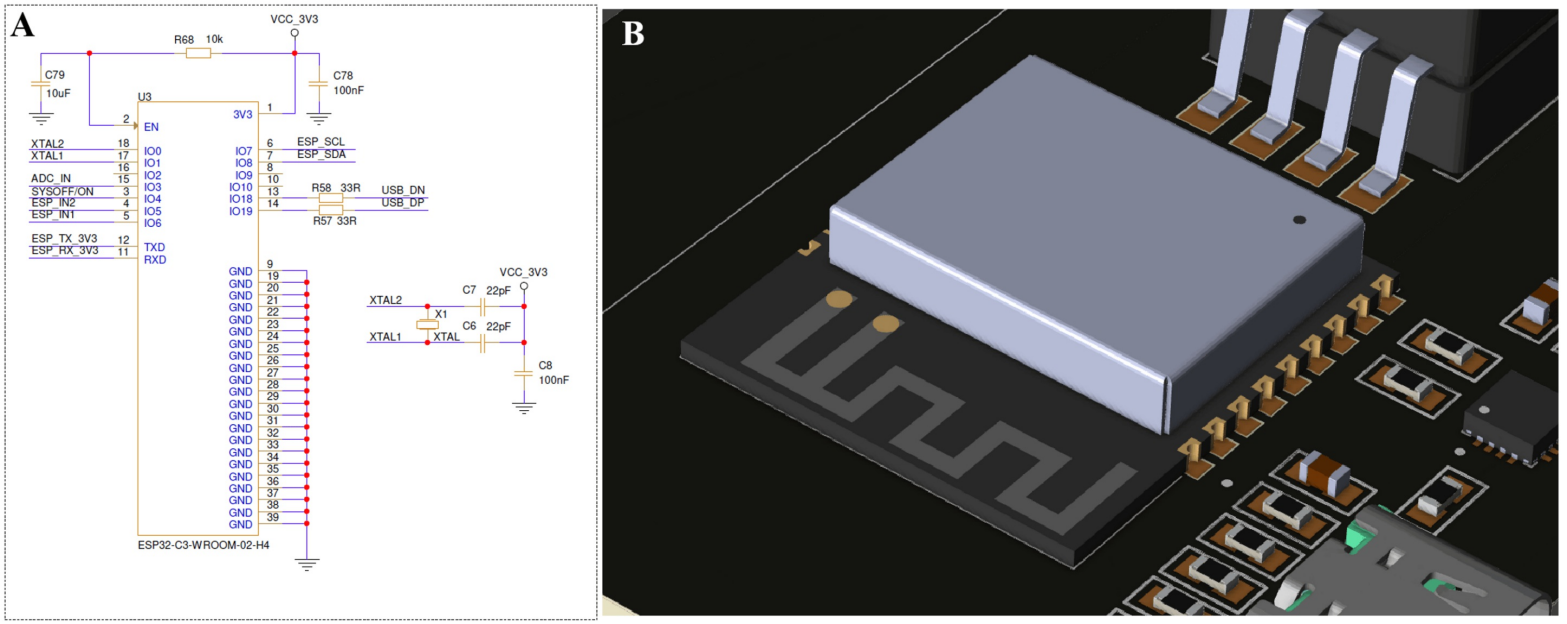
ESP32 microcontroller interface and wireless communication module (A) The ESP32-C3-WROOM-02 embedded microcontroller circuit responsible for system control and Bluetooth connectivity. (B) The corresponding PCB layout with the onboard PCB antenna, ensuring reliable wireless data transmission to a smartphone application for real-time capnography display. PCB: printed circuit board Image Credit: Author's original illustration

The ESP32-C3-WROOM-02 module serves as the primary embedded controller responsible for sensor sampling, pump actuation, and wireless data streaming. As shown in Figure 6, the microcontroller operates at 3.3 V and is stabilized with a 40 MHz crystal oscillator and local decoupling. Universal asynchronous receiver/transmitter (UART) and I²C interfaces support communication with the NDIR CO₂ sensor, pressure sensor, and motor driver, while an integrated 2.4 GHz printed circuit board (PCB) antenna enables low-latency Bluetooth connection to external monitoring devices.

**Figure 7:**
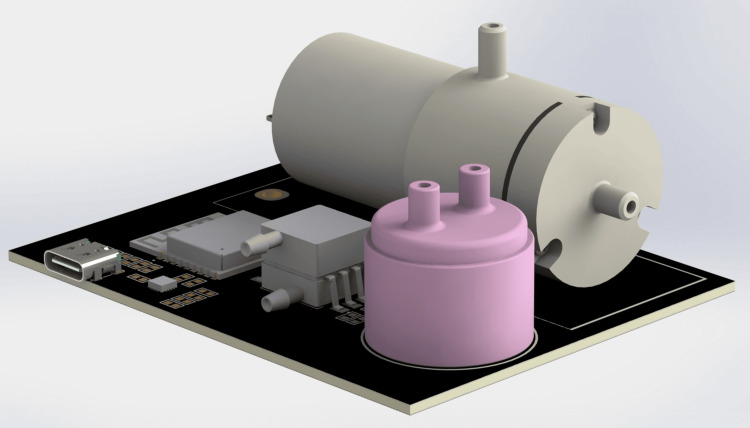
3D rendered prototype of the portable capnography device 3D rendered view of the proposed portable capnography monitoring system, illustrating the compact integration of all major components on a single PCB. PCB: printed circuit board Image Credit: Author's original illustration

Figure 7 illustrates the fully integrated hardware design of the proposed portable capnography device as a compact, PCB-mounted system. The 3D rendering shows the physical arrangement of the NDIR CO₂ sensor, miniature diaphragm pump, and ESP32 communication and control module, enabling continuous airflow sampling and real-time CO₂ analysis within a small form factor. The onboard USB Type-C port supports both power and data transfer, while the surrounding circuitry provides power regulation, sensor signal conditioning, and pump actuation. This compact integration enables a lightweight, portable solution suitable for use in pre-hospital care, patient transport, and home-monitoring environments, where rapid and reliable ventilation assessment is crucial.

**Figure 8:**
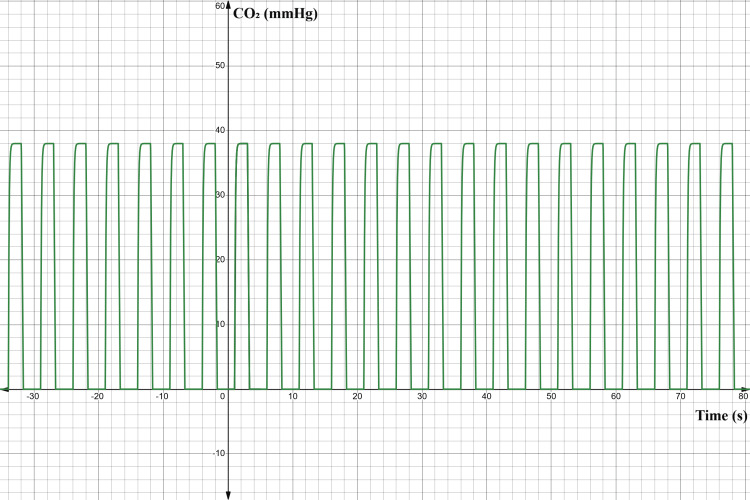
Simulated capnography waveform representing ETCO₂ variation over time A simulated capnography signal was generated to model the expected ETCO₂ dynamics during a normal respiratory cycle. The waveform clearly demonstrates the expected plateau near 38 mmHg, indicating adequate ventilation and gas exchange. CO₂: carbon dioxide; ETCO₂: end-tidal carbon dioxide Image Credit: Author's original illustration

The simulated signal presented in Figure 8 reproduces the characteristic phases of a clinical capnograph, with the y-axis representing CO₂ partial pressure (in mmHg) and the x-axis representing time (s). The waveform peaks near 38 mmHg, corresponding to normal alveolar CO₂ concentration (ETCO₂) in a healthy adult at sea level. Each cycle illustrates the four phases of respiration: baseline (Phase I), expiratory upstroke (Phase II), alveolar plateau (Phase III), and inspiratory downstroke (Phase IV). The baseline near 0 mmHg reflects the inhalation of CO₂-free air, while the plateau indicates the steady exhalation of alveolar gas.

The device is powered by a single-cell lithium battery and generates two regulated voltage rails: 3.3 V for the ESP32 microcontroller, digital interfaces, and sensing electronics and 4.5 V for the miniature pump driver. Both rails are produced using high-efficiency buck-boost regulation with local decoupling to minimize electrical noise on the measurement path. Battery safety features include over-current protection and brownout handling to ensure reliable operation during transient load changes, such as pump activation. Under typical use conditions, the total average system load supports an estimated 22-24 hours of continuous operation on a 2000 mAh lithium cell, enabling full-day monitoring in emergency and home-care environments without frequent recharging.

## Discussion

Portable respiratory monitoring continues to gain importance as respiratory illnesses, home-based care, and pre-hospital emergency interventions increase worldwide. Capnography remains one of the most sensitive methods for evaluating ventilation adequacy, airway integrity, and metabolic status, but existing equipment is typically bulky, expensive, and constrained to hospital environments. The compact portable device presented in this work addresses the need for accessible, point-of-care CO₂ monitoring by integrating a high-performance NDIR CO₂ sensor, an automated gas-purging mechanism, and Bluetooth connectivity into a pocket-sized platform.

The proposed system is designed to provide reliable ETCO₂ waveform acquisition during both routine and emergency conditions. Real-time purging of residual CO₂ between breaths enhances waveform fidelity and reduces rebreathing artifacts that commonly degrade low-flow measurements in portable devices. Wireless data transmission further enables the continuous remote visibility of respiratory status, which is particularly relevant for early deterioration detection, triage in resource-limited settings, and monitoring during patient transport.

Though promising, this work has several limitations that warrant further investigation. Performance testing has not yet been conducted against clinical-grade reference equipment, and the long-term drift behavior of the NDIR sensor remains to be evaluated.

Additional future work will focus on refining enclosure ergonomics, evaluating data transmission reliability during movement, and performing clinical comparisons to validate ETCO₂ accuracy under varying respiratory conditions.

## Conclusions

This work demonstrates the development of a compact, battery-powered portable capnography system designed to extend respiratory monitoring beyond traditional hospital settings. The device supports more than 24 hours of continuous ETCO₂ measurement on a single charge, enabling uninterrupted use during patient transport, prolonged home-care observation, and emergency response where access to standard monitoring equipment is limited. By combining a high-performance NDIR CO₂ sensor, pressure-based breath detection, automated chamber purging, and Bluetooth wireless transmission, the system provides clinically valuable waveform data in a lightweight, pocket-sized platform.
